# No, There Is No 150 ms Lead of Visual Speech on Auditory Speech, but a Range of Audiovisual Asynchronies Varying from Small Audio Lead to Large Audio Lag

**DOI:** 10.1371/journal.pcbi.1003743

**Published:** 2014-07-31

**Authors:** Jean-Luc Schwartz, Christophe Savariaux

**Affiliations:** GIPSA-Lab, Speech and Cognition Department, UMR 5216 CNRS Grenoble-Alps University, Grenoble, France; Cognitive Systems Research Institute, Greece

## Abstract

An increasing number of neuroscience papers capitalize on the assumption published in this journal that visual speech would be typically 150 ms ahead of auditory speech. It happens that the estimation of audiovisual asynchrony in the reference paper is valid only in very specific cases, for isolated consonant-vowel syllables or at the beginning of a speech utterance, in what we call “preparatory gestures”. However, when syllables are chained in sequences, as they are typically in most parts of a natural speech utterance, asynchrony should be defined in a different way. This is what we call “comodulatory gestures” providing auditory and visual events more or less in synchrony. We provide audiovisual data on sequences of plosive-vowel syllables (pa, ta, ka, ba, da, ga, ma, na) showing that audiovisual synchrony is actually rather precise, varying between 20 ms audio lead and 70 ms audio lag. We show how more complex speech material should result in a range typically varying between 40 ms audio lead and 200 ms audio lag, and we discuss how this natural coordination is reflected in the so-called temporal integration window for audiovisual speech perception. Finally we present a toy model of auditory and audiovisual predictive coding, showing that visual lead is actually not necessary for visual prediction.

## Author Summary

Since a paper was published in this journal, an increasing number of neuroscience papers capitalize on the assumption that visual speech would be typically 150 ms ahead of auditory speech. It happens that the estimation of audiovisual asynchrony in the mentioned paper is valid only in very specific cases, for isolated consonant-vowel syllables or at the beginning of a speech utterance. But the view that vision leads audition is globally oversimplified and often wrong. It should be replaced by the acknowledgement that the temporal relationship between auditory and visual cues is complex, including a range of configurations more or less reflected by the temporal integration window from 30 to 50 ms auditory lead to 170 to 200 ms visual lead. This has important consequences for computational models of audiovisual speech processing in the human brain.

## Introduction

### Early audiovisual interactions in the human brain

Sensory processing has long been conceived as modular and hierarchic, beginning by monosensory cue extraction in the primary sensory cortices before higher level multisensory interactions took place in associative areas, preparing the route for final decision and adequate behavioral answer. However, it is now firmly established that low-level multisensory interactions are much more pervasive than classical views assumed they were and affect brain regions and neural responses traditionally considered as modality specific [1], [2].

Restricting to audiovisual interactions in speech perception, direct connections have been displayed between primary auditory cortex and primary visual cortex (e.g. [3] on macaques), and electrophysiological data on speech perception display early influence of the visual component of speech stimuli on auditory evoked response potentials (ERPs). Indeed, there appears a decrease in amplitude and latency of the first negative peak N1 and the second positive peak P2, 100 to 200 ms after the acoustic onset, when the visual component is present [4], [5]. It is still under debate to determine the specific role of direct connections between primary sensory cortices vs. the role of associative cortex and particularly the superior temporal sulcus in these early interactions [6]–[8].

The computational nature of audiovisual interactions is now the focus of a large number of recent papers. Capitalizing on the natural rhythmicity of the auditory speech input, it has been suggested [9], [10] that the visual input could enhance neuronal oscillations thanks to a phase-resetting mechanism across sensory modalities. This has led to various experimental demonstrations that visual speech improves the tracking of audiovisual speech information in the auditory cortex by phase coupling of auditory and visual cortices [11], [12].

A number of these studies have proposed predictive coding as a possible unifying framework for dealing with audiovisual interactions. Predictive coding posits that neural processing exploits a differential coding between predicted and incoming signals, with decreased activity when a signal is correctly predicted [13], [14]. Visual prediction would be responsible for early modifications in auditory ERPs evoked by visual speech decreasing latency and amplitude of N1 and P2 (e.g. [5], [7]). This has led to recent proposals about the role of specific components in neural oscillations respectively conveying top-down predictions and bottom-up prediction errors in audiovisual speech processing [8], [15].

### The underlying audiovisual structure of speech stimuli

The previously mentioned studies capitalize on the underlying audiovisual structure of speech stimuli, that is the way sounds and sights provided by the speaker are comodulated in time (so that their phase can indeed be coupled) and more generally how one modality provides adequate information for partial prediction of the other modality.

It is actually known since long that the auditory and video streams are related by a high level of cross-predictability related to their common underlying motor cause. This is displayed in a number of studies about audio-visual correlations between various kinds of video (e.g. lip parameters, facial flesh points, video features extracted from the face) and audio (acoustic envelope, band-pass filter outputs, spectral features) parameters [16]–[20].

In a recent and influential paper published in this journal, Chandrasekaran et al. [21] present a number of analyses about the “natural statistics of audiovisual speech”, based on various databases in different languages (British and American English, and French), with four major results: firstly, there is a robust correlation in time between variations of mouth opening and variations of the acoustic envelope; secondly, focusing the acoustic envelope to narrow regions in the acoustic spectrum, correlation is maximum in two regions, one around 300–800 Hz, typically where is situated the first vocal tract resonance (formant) F1, and the other around 3000 Hz interpreted by the authors as corresponding to the second and third resonances F2 and F3; thirdly, temporal comodulations of the mouth and acoustic envelope appear in the 2–7 Hz frequency range, typically corresponding to the syllabic rhythm; last but not least in the context of the present paper, “the timing of mouth movements relative to the onset of the voice is consistently between 100 and 300 ms” (penultimate sentence of the paper abstract).

Since the publication of this paper and systematically referring to it, an increasing number of neuroscience papers – including some of those cited previously – capitalize on the assumption that visual speech would be typically 150 ms ahead of auditory speech. Let us mention a few quotations from these papers: “In most ecological settings, auditory input lags visual input, i.e., mouth movements and speech associated gestures, by ∼150 ms” [7], [8]; “there is a typical visual to auditory lag of 150–200 ms in face-to-face communication” [22]; “articulatory facial movements are also correlated with the speech envelope and precede it by ∼150 ms” [12].

The invoked natural audiovisual asynchrony is used in these papers in support to development on models and experiments assessing the predictive coding theory. The assumption that image leads sound plays two different roles in the above mentioned neuroscience papers. It is sometimes used as a trick to demonstrate that the visual stimulus plays a role in modulating the neural auditory response, rightly capitalizing on a situation where a consonant-vowel (CV) sequence (e.g. “pa” or “ta”) is produced after a pause. In this case, the preparatory movement of the mouth and lips is visible before any sound is produced, hence visual prediction can occur ahead of sound and results in visual modulation of auditory ERPs [4], [5], [7].

The second role is more problematic. Considering that there would be a systematic and more or less stable advance of vision on audition around 150 ms, it is proposed that this situation would play a role in the ability to use the visual input to predict the auditory one all along the time. Audiovisual asynchrony is implicitly incorporated in a number of models and proposals.

However, as we will see in the next section, the situation studied in [21] is very specific, characteristic of a CV sequence produced in isolation or at the beginning of an utterance after a pause. The objective of the present paper is to show that, while the method proposed by Chandrasekaran et al. to estimate audiovisual delays is adequate for the onset in preparatory sequences or the start of a speech utterance, in chained sequences which actually provide the most general case in speech communication, the method should be modified. Furthermore, if an appropriate method is used, delays actually vary in a different range from the one they propose – with the consequence that “there is no 150 ms lead of visual speech on auditory speech”.

### Preparatory gestures and comodulatory gestures: The hammer and the balloon

**Figure 1:**
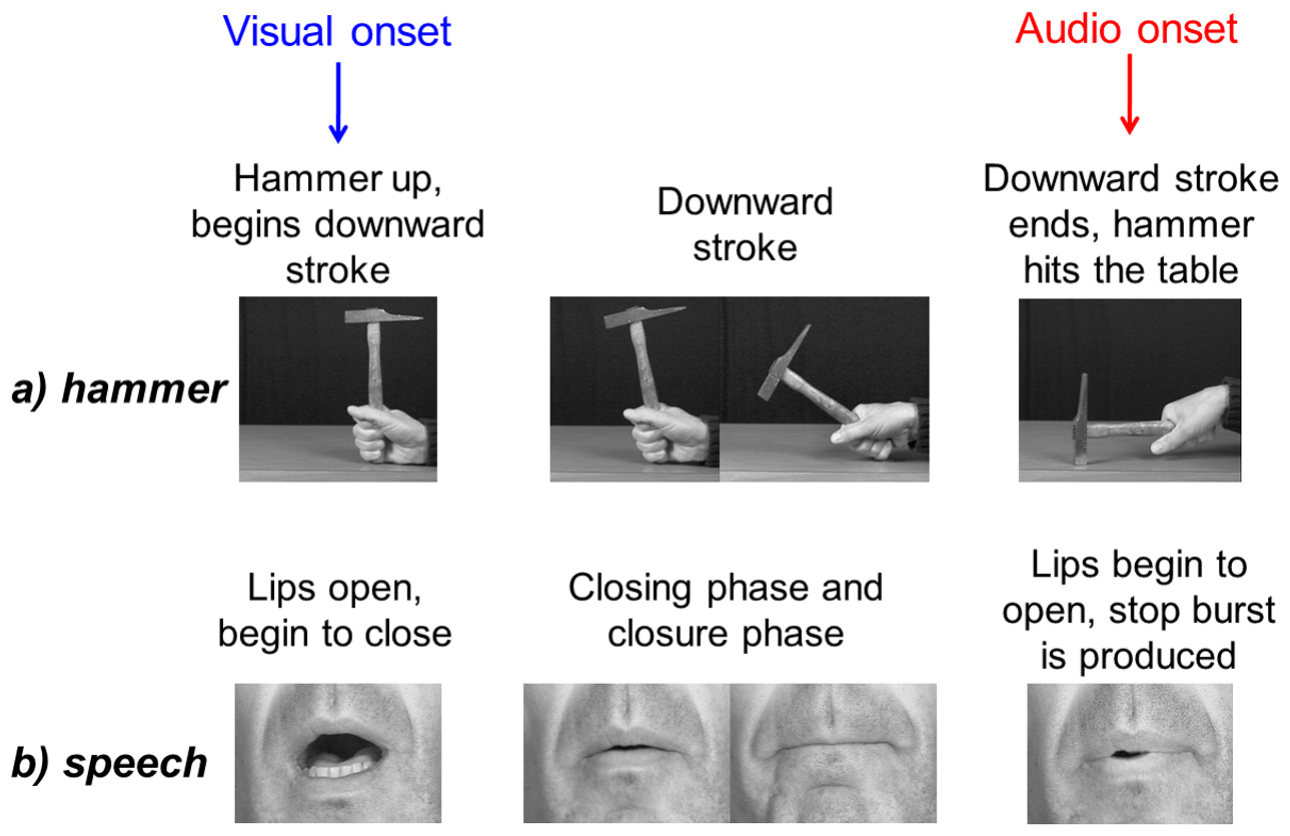
Preparatory gestures are visible and not audible. (a) A preparatory gesture for a hammer hitting a table. (b) A preparatory gesture for a labial burst after a pause.

The rationale in the measure of asynchrony proposed by Chandrasekaran et al. is based on the notion of *preparatory gestures* (Figure 1). This is also the case of the N1-P2 studies mentioned previously (e.g. [5], [8]). This can be related to a rather classical analogy, namely the movement of a hammer towards a table (Figure 1a). To produce a sound with a hammer, one must previously realize a downward stroke and the onset of this downward stroke is visible much before the hammer touches the table and makes a sound. Notice that in this scene, one could define actually *two* visible events, one at the onset of the downward stroke and one at the instant when the hammer touches the table; and only *one* auditory event, the sound onset, which is actually perfectly synchronous with the second visual event. The downward stroke may be called a “preparatory gesture” in that it prepares the sound and hence predicts something about it (its time of arrival, and also its acoustic content since a subject looking at the hammer going towards the table knows the kind of sound which will be produced soon).

It is exactly the same for preparatory lip gestures before “p” at the beginning of a speech utterance (Figure 1b): when the lips begin to close, a subject looking at the speaker knows that they will soon join together for a lip closure, and she/he can predict rather accurately when will sound occur and what will be its spectrum (the typical flat low-frequency spectrum of a bilabial burst [23]). Here again, there are two visual events, namely the onset of the lip closing gesture and the further onset of the lip opening gesture, and only one auditory event, the burst onset, quite synchronous with the second visual event. Notice that the analogy between the preparatory gestures for the hammer and for speech is not perfect. Indeed, the sound is produced by the hammer at the end of the downward stroke, while for speech the lips must open again. There is actually a complex coordination between larynx, lungs and lips to achieve the adequate aerodynamic strategy [24], which fixes rules about the duration of lip closure before lip opening. But the audiovisual asynchrony involved in preparatory gestures for both hammer and speech are similar: in both cases, audiovisual asynchrony is assessed by the duration between two *different* events, the onset of the preparatory gesture for the visual channel and its offset for the auditory channel.

Therefore it appears that the crucial aspect of preparatory gestures is that they are visible but produce no sound. This could be different, actually. Consider for example what happens if you replace the hammer by a whip or a flexible stick. Now the downward stroke produces a whistling sound (which also predicts the sound produced when the whip or stick touches the table). There are now *two* auditory events, just as there are two visual events, and for both pairs of audiovisual events (at the beginning and end of the visual stroke) the auditory and visual events are quite in synchrony.

**Figure 2:**
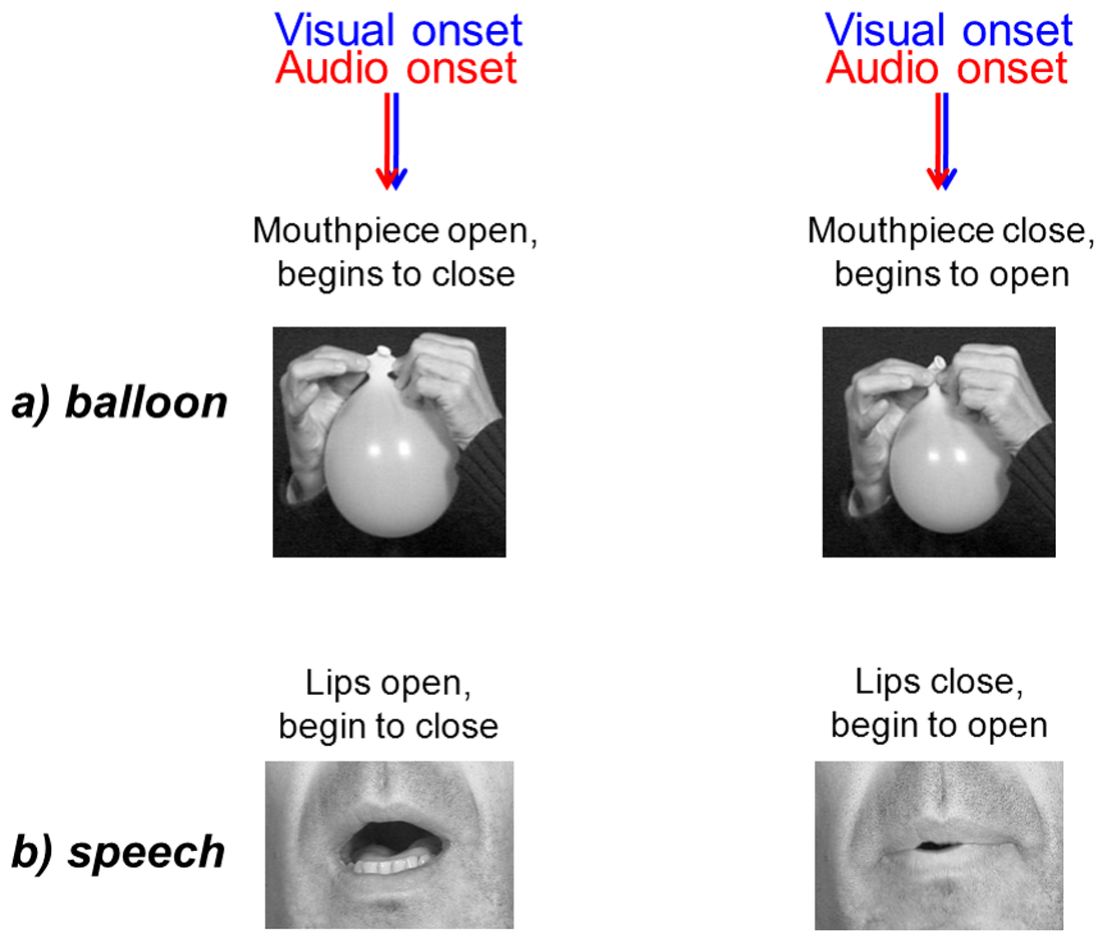
Comodulatory gestures are visible and audible. (a) A comodulatory closing/opening gesture for a balloon. (b) A comodulatory closing/opening gesture for lips in speech communication.

This leads us towards another kind of gestures that we propose to call “comodulatory gestures” since these gestures produce both auditory and visual stimuli more or less in synchrony all along the time (Figure 2). Comodulatory gestures are actually by far the most common gestures in speech. Here we should move towards another analogy that is a balloon in which one adjusts the mouthpiece. When its size increases or decreases, shape, volume and pressure change leading to more or less synchronous auditory and visual events for both opening and closing phases (Figure 2a), just as opening and closing the lips while vocalizing produces auditory and visible events quite in synchrony (Figure 2b).

### Objectives of this paper

In the remaining of this paper we present simple audiovisual data on plosive-vowel syllables (pa, ta, ka, ba, da, ga, ma, na), produced either in isolation or in sequence. We show that when syllables are produced in isolation, preparatory gestures provide audiovisual asynchronies quite in line with those measured in [21]. However, when syllables are chained in sequences, they provide comodulatory gestures in which audiovisual synchrony is actually precise, contrary to the data provided on similar sequences in [21], just because the measure of audiovisual asynchrony is different. In such cases, there are actually auditory events that were not taken into account in the original paper, and these need to be taken into account if one is talking about asynchrony.

After presenting Methodology and Results, we discuss how natural coordination between sound and image can actually produce both cases of lead and lag of the visual input. We relate the range of leads and lags to the so-called temporal integration window for audiovisual speech perception [25]. We propose that the “visual lead” hypothesis, wrong in many cases, is actually not necessary to deal with audiovisual predictability, and we illustrate this by briefly introducing a simple audiovisual prediction model dealing with the speech sequences studied previously. We conclude by some methodological and theoretical remarks on neurophysiological developments about audiovisual predictability in the human brain.

## Methods

### Data

In the experimental work we focus on audiovisual temporal relationships in CV sequences where C is a voiced, unvoiced or nasal stop consonant that is, for English or French (the two languages considered in [21]), one of the sounds /p t k b d g m n/, and V is the open vowel /a/. We consider both CV sequences produced in isolation and chained sequences VCVCVCV. This corpus is very simple though sufficient to illustrate the difference between preparatory gestures – for isolated syllables – and comodulatory gestures – for chained syllables. The /a/ context in which the plosives /p t k b d g m n/ are produced is selected because it provides a large gesture amplitude providing more salient trajectories both in the visual and auditory modality. We will consider more general phonetic material in the discussion.

We recorded a small database of 6 repetitions of 8 syllables /pa ta ka ba da ga ma na/ uttered by a French speaker either in isolation /Ca/ or in sequence /aCa/. The syllables were produced in a fixed order at a relatively slow rhythm (around 800 ms per syllable). In the “isolated syllables” condition, syllables were embedded in silence: /pa#ta#ka#ba#da#ga#ma#na/ where /#/ means a silence (typically 500 ms silence between two consecutive syllables). In the “chained syllables” condition, they were produced in the same order though with no silence between syllables: /apatakabadagamana/.

The recording was done with a PAL camera at 50 Hz. The recording set up was based on the classical paradigm we use in Grenoble since years [26], [27] with blue make up applied on the lips. For each image, we extracted automatically and precisely the lip contours by applying a Chroma Key process extracting blue areas on the face. The lips parameters were extracted every 20 ms, synchronously with the acoustic signal, which is sampled at 22.05 kHz.

### Analysis

#### Detection of auditory and visual events

Then on each CV utterance of this database we labeled auditory and visual events.

**Figure 3:**
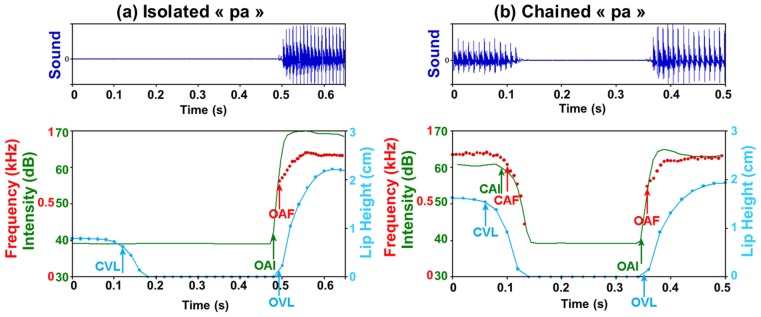
Acoustic signal (top panel), intensity in green, lip height in blue and formants in red (bottom panel): For isolated /pa/ (a, left) and /apa/ (b, right). Blue arrows: lip events. Green arrows: intensity events. Red arrows: formant events. CAF/OAF (red): Closing/Opening onset for Audio Formant. CAI/OAI (green): Closing/Opening onset for Audio Intensity. CVL/OVL (blue): Closing/Opening onset for Visible Lips.

The acoustic analysis was done with Praat [28]. The first formant was extracted after a Linear Predictive Coding (LPC) analysis. A typical display of the synchronized acoustic signal with its time-frequency analysis (including intensity and formants) and lip trajectory (one measure every 20 ms) is presented on Figure 3a for an isolated /pa/ and Figure 3b for a /pa/ chained in a sequence (with a zoom around the consonant /p/ in /apa/).

On such kinds of displays we detected auditory and visual events corresponding to the onset of opening or closing gestures, with criteria based on given ranges of energy decrease/increase – 1 dB –, formant decrease/increase – 60 Hz – or lip height decrease/increase – 0.15 cm – from previous minimal or maximal values. For the detection of visual events, considering the rather low sampling frequency at 50 Hz and since lip opening may be rather quick, specifically for bilabials, we applied linear interpolation between lip height values at two consecutive images to refine event detection. We labelled the corresponding events:

on the acoustic signal, in the case of chained sequences (Figure 3b): the beginning of the decrease of the first formant F1 in the portion from the previous “a” to the next plosive (Closing onset for Audio Formant: CAF); the corresponding beginning of intensity decrease (Closing onset for Audio Intensity: CAI). And in all cases, for chained as well as isolated sequences, the beginning of F1 increase in the portion from the plosive to the next “a” (Opening onset for Audio Formant: OAF) and the corresponding beginning of intensity increase, that is the burst onset (Opening onset for Audio Intensity: OAI).
on the lip trajectory, in all cases: the beginning of lip area decrease in the portion from the previous “a” or from silence to the next plosive (Closing onset for Visible Lips: CVL) and the beginning of lip area increase at the plosive release towards the next vowel (Opening onset for Visible Lips: OVL).

#### Estimation of audiovisual asynchrony

Estimation of audiovisual temporal relationship is done differently for preparatory gestures (isolated sequences) and comodulatory gestures (chained sequences).

**Figure 4:**
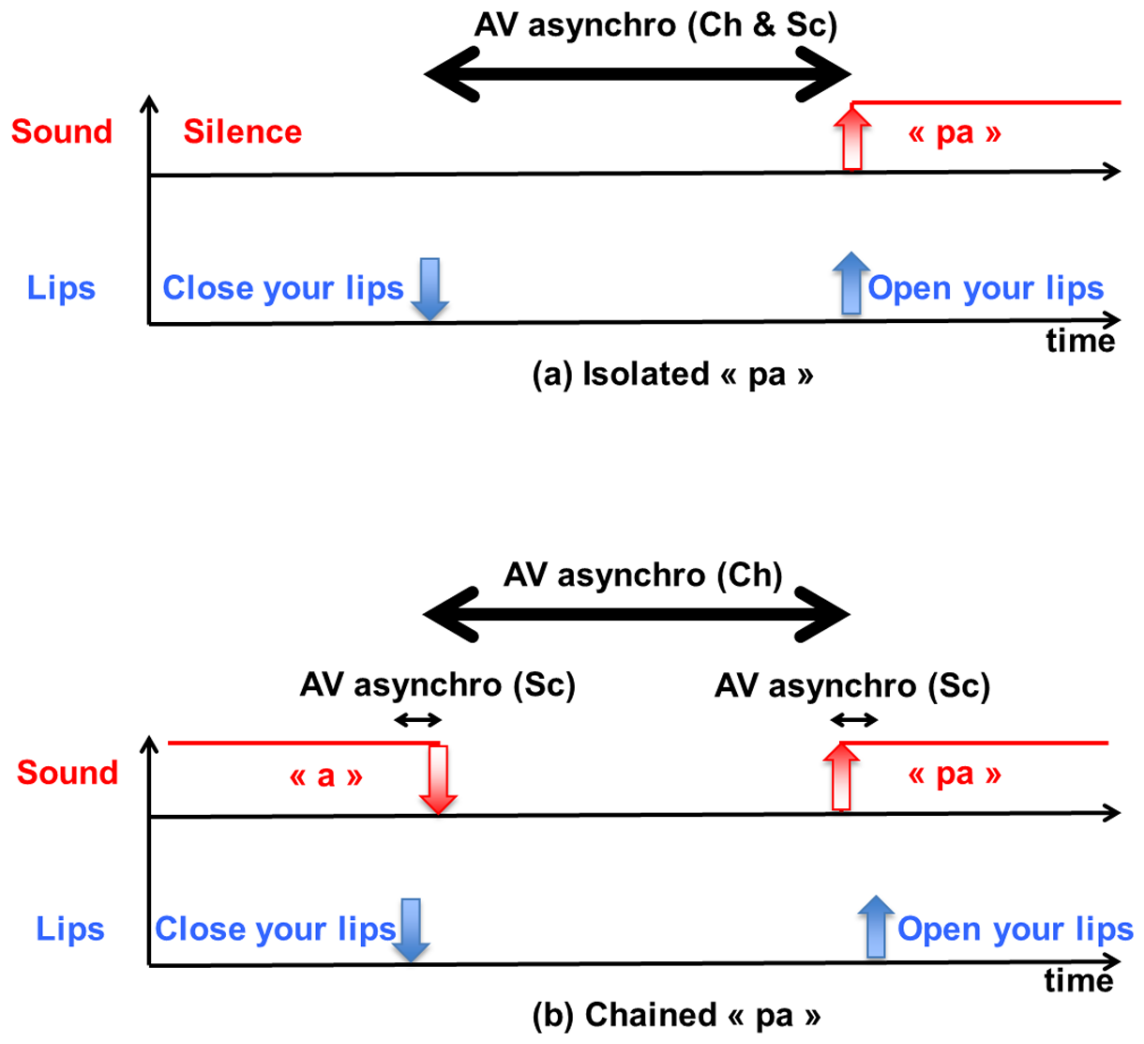
Sequence of auditory and visual events and measure of audiovisual asynchrony in isolated “pa” (top) and “pa” chained in a sequence “apa” (bottom). AV asynchro (Ch) refers to the AV asynchrony measure used in [1], AV asynchro (Sc) refers to the AV asynchrony measure used in the present paper.

For isolated syllables such as /pa/ (Figure 4a), lips first close to prepare the “p”. This involves a visible gesture described in [21] by two temporal events, the initiation of the closing gesture, and the velocity peak of the lips during the closure phase (down blue arrow in Figure 4a). Then comes the release, which corresponds to a third visible event that is an opening onset (up blue arrow in Figure 4a, not discussed by the authors) and to the first auditory event that is the acoustic burst for the plosive (up red arrow in Figure 4a). Of course, the first visible event (closure gesture initiation, down blue arrow) and the first auditory event (opening gesture initiation, up red arrow) are asynchronous, since closure must occur before opening. Asynchrony is described in this case between the first visible event and the first auditory event, providing the same measure in our study (AV asynchro (Sc) in Figure 4, with Sc for Schwartz and Savariaux) and in Chandrasekaran et al. (AV asynchro (Ch)). The temporal distance may reach 150 ms or even more: actually lips can close any time before they open (imagine you want to stop your interlocutor by uttering “please”, you prepare the “p” but don't succeed to interrupt him or her: you will stay with your lips closed for a while, and the temporal delay between visible lip closing and audible burst may reach very large values).

For chained sequences such as “apa” (Figure 4b), lips closure is both visible (down blue arrow in Figure 4b) and audible since it changes the formants (acoustic resonances of the vocal tract) and the intensity of the sound (down red arrow in Figure 4b). At the end of the closing gesture the sound stops (or changes into intervocalic voicing in the case of “aba”). In such cases it is mistaken to characterize audiovisual coordination as the delay between closing gesture initiation for vision (down blue arrow) and opening gesture initiation for audition (up red arrow) – though this is what Chandrasekaran et al. do in their Figure 9 – because there is actually an audible and a visible event for both closure gesture (down blue and red arrows) and opening gesture initiation (up blue and red arrows). This provides therefore different measures of asynchrony in our study and in [21].

Altogether this results in completely different estimations of audiovisual asynchrony for preparatory gestures (Figure 4a) and comodulatory gestures (Figure 4b). Of course one could argue that it is better to use the same measure for asynchrony in all situations, but the measure used in [21] in the case of chained sequences – actually corresponding to what happens in most of continuous speech – is inappropriate since it forgets audible events in the closing phase.

## Results

### Isolated syllables: Confirming [21] for preparatory gestures

**Figure 5:**
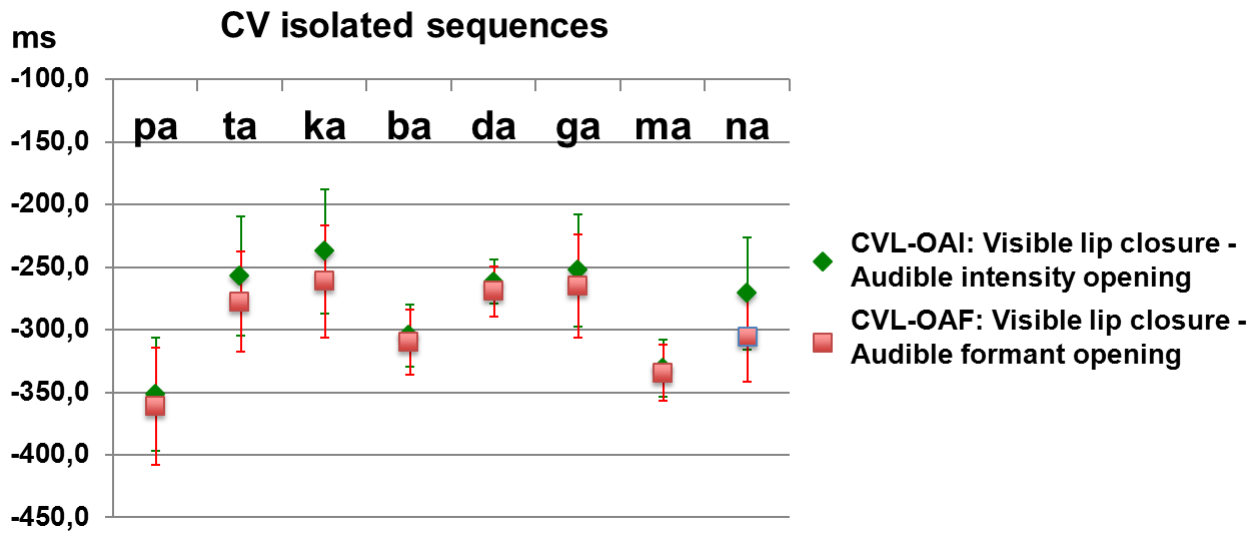
Delay between the first visual event (for the closing phase) and the first auditory event (for the opening phase) in isolated /Ca/. Negative values mean that the acoustic event lags the visual one. In red: acoustic events for formants. In green: acoustic events for intensity. Signs point at mean values (over the 6 repetitions), and error bars correspond to the standard deviation.

We display on Figure 5 the data for isolated syllables. In this case, where there is no audible event for closure, we report the same measure as in [21] that is the delay between the first visible event, CVL, and the first audible event, OAI or OAF. There is a very large anticipation, which actually reaches values much larger than 150 ms here (and which may reach 400 ms in some cases). These values are compatible with the range 100-300 ms proposed in [21], the more so considering that the measure used by the authors for detecting visual events (half open point in the lip closing trajectory, while we used the onset of the closing phase) would produce values lower than the ones in Figure 5.

### Chained syllables: Infirming [21] for comodulatory gestures

**Figure 6:**
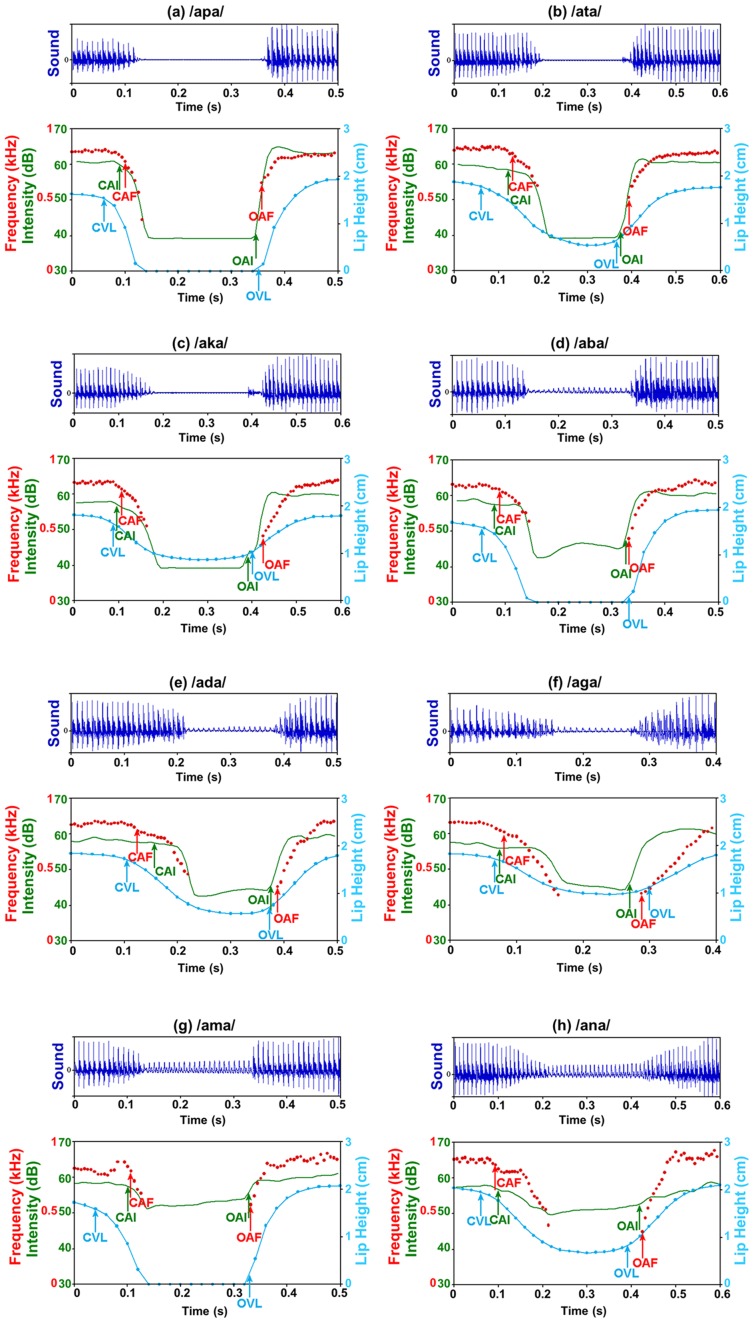
Acoustic signal (top panel), intensity in green, lip height in blue and formants in red for the 8 chained sequences. Blue arrows: lip events (CVL/OVL: Closing/Opening onset for Visible Lips). Green arrows: intensity events (CAI/OAI: Closing/Opening onset for Audio Intensity). Red arrows: formant events (CAF/OAF: Closing/Opening onset for Audio Formant). (a) /apa/; (b) /ata/; (c) /aka/; (d) /aba/; (e) /ada/; (f) /aga/; (g) /ama/; (h) /ana/.

We display on Figure 6 typical audiovisual sequences for all types of chained syllables (with a zoom around the consonant). It clearly shows that there is comodulation of the auditory and visual information, with audible and visible events for both closing and opening phases. The event detection is sometimes not straightforward or not very precise in time (e.g. detection of CAI for /ata/ or /ada/), which is quite classical in this type of stimuli, and gross trends are more important that precise values in the following.

**Figure 7:**
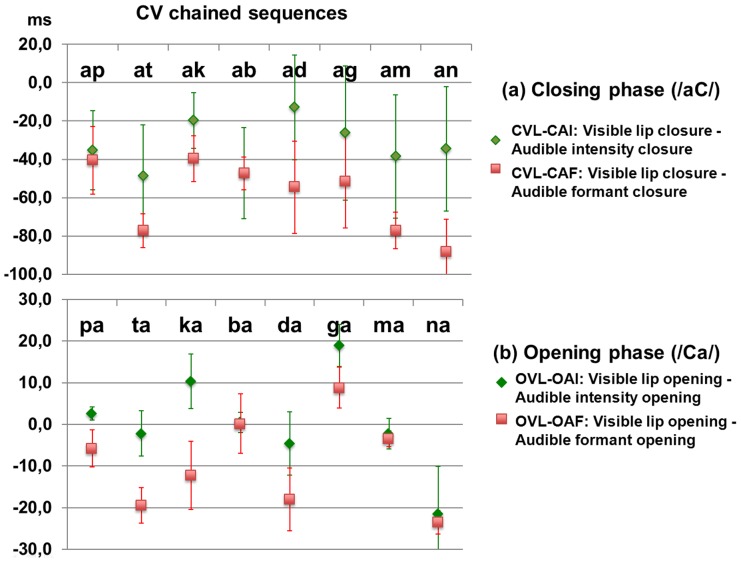
Delay between visual and auditory events: (a) in the closing phase, in /aC/ where C is a plosive in the set /p t k b d g m n/; (b) in the opening phase, in /Ca/ with the same plosives. Positive values mean that the acoustic event leads the visual one. In red: acoustic events for formants. In green: acoustic events for intensity. Signs point at mean values (over the 6 repetitions), and error bars correspond to the standard deviation.

We display on Figure 7 the data about temporal coordination between audio and visual events for either closing (Figure 7a) or opening (Figure 7b) in the case of chained sequences. The mean delay between visual and acoustic events at the closure (in the /aC/ portion, Figure 7a) varies between −20 ms and −40 ms for intensity (CVL-CAI, in green) and reaches values from −40 to −80 ms for formants (CVL-CAF, in red). This means that there is a small lead of the visual channel compared to the audio channel (where information is available on intensity before formants). But this lead is much smaller than the 150 ms lead mentioned in [21], and there are actually cases where audio and video information are available more or less in synchrony, e.g. for /ad/, /ag/ or /ak/ where the tongue gesture towards the voiced plosive decreases intensity or formants while jaw may stay rather stable, and hence lip area does not decrease much – which prevents early video detection.

In the opening phase (Figure 7b) the synchrony is even larger. Concentrating on the delay between labial and intensity events (OVL-OAI, in green) we actually observe an almost perfect synchrony for labials (/p b m/). This is trivial: as soon as the lips begin to open, the sound drastically changes, from silence (for /p/) or prevoicing (for /b/) or nasal murmur (for /m/) to the plosive burst. For velars /k g/ there is actually a clear lead of the audio channel, since the first tongue movement producing the plosive release is done with no jaw movement at all and hence before any labial event is actually detectable: the audio lead may reach more than 20 ms (see examples in Figure 6). Notice that while the video sampling frequency at 50 Hz can make the detection of the opening event for bilabials a bit imprecise with a precision around 10 ms for very quick gestures, the variations of lip area for dentals or velars is smooth and hence imprecision in event detection cannot explain such an audio lead.

Therefore the discrepancy with [21] is clear for chained syllables, just because this corresponds to what we called comodulatory gestures, for which we argue that a different measure of the audiovisual asynchrony should be used.

## Discussion

### Summary of the experimental results

The experimental results presented previously show that for isolated syllables associated with preparatory gestures, our measure of audiovisual asynchrony provides quantitative estimates from 200 ms to 400 ms of visual lead (Figure 5). This is in line with the 100 to 300 ms visual lead proposed in [21], the more so considering that the estimate of the visible onset for lip closure in [21] is done at the mid closing phase – while we prefer detecting the first visible event that is at the very beginning of the lip closure phase, typically 100 ms before. The coherence of both sets of measures was expected considering that the same definition of asynchrony for preparatory gestures is used in both papers, between the first visible event (onset of lip closing phase) and the first auditory event (plosive burst at labial release).

However the data are quite different for chained sequences associated with comodulatory gestures. In this case the range of asynchronies is much more restricted and more centered around 0, from 70 ms visual lead to 20 ms audio lead when auditory events are detected on intensity, auditory events detected on the formant trajectory being somewhat delayed in respect to intensity (Figure 7). Mean video lead amounts to 35 ms in the closing phase and 0 ms in the opening phase for intensity, 60 ms in the closing phase and less than 10 ms in the opening phase for formants. Therefore the departure between our data and those proposed in [21] is now important. This is not due to variability in the speech material, but to a difference in the measure proposed for assessing audiovisual asynchrony. As explained in Figure 4, the measures differ hence their results also differ. Speech gestures in chained sequences typically produce both auditory and visual events all along the time (see Figure 6) hence resulting in a rather precise audiovisual synchrony in most cases.

### The range of possible AV asynchronies in human speech

Preparatory gestures do exist in speech communication, and ERP studies rightly capitalized on this experimental situation in which the gap between the first visible and the first auditory event may be quite large and able to lead to significant influence of the visual input on the electrophysiological response in the auditory cortex, for both speech [5], [8] and non-speech stimuli [29], [30]. Notice that this may actually depend on the prephonatory configuration: if somebody keeps the lips closed while listening to the interlocutor, there will actually be no preparatory gesture before an initial bilabial sound such as /b/ or /m/, and hence there will be no visual lead at all in this case. One could even imagine a reverse situation in which a speaker keeps the lips closed and systematically signals her/his turn taking by a backchannel signal “hmm” (which is not rare): in this case the preparatory gesture would be actually audible and not visible, leading to an auditory lead in the preparatory phase.

However, most of the speech material is made of comodulatory gestures. Of course, speech utterances involve a range of phonetic configurations much larger than the /Ca/ sequences that were studied in this paper. This variety of configurations leads to a variety of situations in terms of audiovisual asynchronies. This is where the analogy we proposed previously with the deflating balloon being both audible and visible reaches some limits: actually, not every action realized on the vocal tract is always either audible or visible, which may lead to delays between perceivable auditory or visible cues for a given speech gesture.

A first general property of speech concerns anticipatory coarticulation – much more relevant and general than preparatory movements discussed in [21]. This relates to articulatory gestures towards a given phonetic target, which can begin within a previous phoneme. Anticipatory coarticulation generally capitalizes on a property of the articulatory-to-acoustic transform, in which an articulatory gesture has sometimes no or weak effect on the sound and hence can be prepared in advance without audible consequences.

A typical example concerns the rounding gesture from /i/ to /y/ or /u/ in sequences such as /iC_1_C_2_…C_n_y/ or /iC_1_C_2_…C_n_u/ with a variable number of consonants C_1_…C_n_ not involving a specific labial control (e.g. /s t k r/) between the unrounded /i/ and the rounded /y/ or /u/. In this case the rounding gesture from /i/ towards /y/ or /u/ can begin within the sequence of consonants /C_1_C_2_…C_n_/, and hence anticipate the vowel by 100 to 300 ms [31]. Various sets of data and various theoretical models of this anticipatory coarticulation process have been proposed in the literature [32]–[36]. In such cases the rounding gesture can hence be visible well before it is audible.

So there are cases where visible information is available before auditory information (e.g. in /iC_1_…C_n_u/ sequences), others where vision and audition are quite synchronous (e.g. in /aCa/ sequences), and there are also cases where audition may actually lead vision as was shown e.g. in Figure 7. But the next question is to know if the auditory and visual systems are able to process the information efficiently as soon as it is available. This is actually not always the case, and in gating experiments on the visual vs. auditory identification of coarticulated sequences, Troille et al. [37] display in some configurations a lead of audition on vision which can reach up to 40 ms, because of the poor visibility of some articulatory gestures. This leads the authors to claim that they have discovered a case where “speech can be heard before it is seen”.

In summary, there are actually a variety of situations from audio lead (estimated to 40 ms in [37]) to visual lead (which can reach more than 200 ms). In their study of mutual information between audio and video parameters on speech sequences, Feldhoffer et al. [38] show that mutual information is maximal for some audio and video parameters when it incorporates a video lead up to 100 ms. In audiovisual speech recognition experiments, Czap [39] obtains a smaller value, recognition scores being higher with a small global video lead (20 ms). Altogether, these global estimations are concordant with the classical view that “in average, the visual stream may lead the auditory stream”, which is generally advocated by specialists of audiovisual speech perception (e.g. [40], [41]). However, the “average” view hides a large range of variations, typically inside a window between 40 ms audio lead to 200 ms visual lead in the phonetic content of normal speech communication.

### Plausible consequences for the temporal integration window for AV speech in the human brain

A large number of recent studies have attempted to characterize the temporal integration window in various kinds of multisensory interactions. This typically involves two kinds of paradigms. Firstly, evaluation of intersensory synchrony may be based on either simultaneity or temporal order judgment tasks (see a recent review in [42]). Secondly, the “multisensory temporal binding window” describes the range of asynchronies between two modalities in which a fused percept may emerge [43].

The “audiovisual temporal integration window” is well described for speech perception (e.g. [44], [45]). Van Wassenhove et al. [25] compared estimates of audiovisual temporal integration window based on either simultaneity perceptual judgments or regions where the McGurk effect seems to stay at a maximal value. They show that these various estimates converge on an asymmetric window between about 30 ms audio lead and 170 ms audio lag.

This provides a set of values rather coherent with the range of possible asynchronies in the speech material itself. Small audio leads may occur because of the lack of visibility of certain audible gestures, as shown in Figure 7 or in gating experiments [37]. Large video leads are mostly due to labial anticipatory coarticulation and described in many studies [31]–[36]. A tentative interpretation is that the perceptual system has internalized this range through a learning process. This is in line with the so-called “unity assumption” [46] according to which subjects would naturally bind together multisensory stimuli referring to a common cause, which would lead to both fused percepts and decreased ability to detect temporal asynchronies [47]. We speculate that unity assumption is based on a statistical learning of the comodulation properties of the auditory and visual streams in the speech natural environment, naturally providing an asymmetrical window around the range [−30 ms, +170 ms].

The asymmetry of the temporal integration window has been the topic of much discussion – including assumptions about the difference between optic and acoustic wave speeds, which cannot however explain such a large asymmetry: a speaker 10 m apart from a listener would not provide more than 30 ms visual advance! We argue here that the psychophysical asymmetry just mirrors the natural phonetic asymmetry, according to which there are plenty of cases of large visual anticipation due to coarticulation – typically in the 100 to 200 ms range – and less cases of auditory anticipation, in a smaller range – typically less than 40 ms as displayed in our data in Figure 7 or in gating data [47]. But, once again, this does not mean that there is a constant visual lead, but rather a range of audiovisual asynchronies mirrored in the temporal integration window.

Recent data on the development of the audiovisual temporal integration window fit rather well with this proposal. Indeed, these data show that the window is initially quite large and then progressively refined by “perceptual narrowing” in the first months of life [48]. The window actually appears to stay rather wide and symmetrical until at least 11 years of age [49]. It is only after this age that the left part of the window (for auditory lead) refines from 200 ms to 100 ms, which is proposed by the authors as the typical value for adults (the fact that these values are larger than in [25] likely comes from the use of a different criterion to define binding windows from simultaneity curves). On the contrary, the right part of the window stays stable. The interpretation is that the large initial symmetric window [−200 ms, +200 ms] is progressively tuned to the window characteristic of the speech input, asymmetric in nature. The fact that learning the asymmetrical pattern occurs so late may appear surprising, but it is in fact compatible with data showing that the maturation of the McGurk effect is not complete before at least 8 years of age for native stimuli and even later for non-native stimuli [50].

There is also a rather large deal of variations of audiovisual temporal integration window from one subject to another [43]. These variations respect the asymmetry trend, though with large variations in quantitative values. The fact that these variations are correlated with the results of various fusion paradigms suggests that inter-individual differences could be related with specific weights attributed by subjects to one or the other modality [51], [52]. Interestingly, it also appears a large ability to tune and decrease the integration window with auditory or visual experience [53], [54], including the possibility to decrease the asymmetry and specifically decrease the large visual-lead part of the window, which suggests that the integration window actually combines stimulus-driven content with individually-tuned perceptual experience.

### AV predictability without AV asynchrony

The data recalled in the previous section rule out over-simplistic claims about audiovisual predictability. Does it raise a problem for predictability in general? The answer is clearly *no*. The reason is that predictability does *not* require asynchrony. Actually, a pure auditory trajectory may provide predictions on its future stages, and the visual input may enhance these predictions, since it is naturally in advance on *future* auditory events, though not systematically in advance on *present* ones. This is illustrated on the toy model presented in [55] and sketchily introduced here under (see a detailed presentation in the Supplementary Text S1).

The model was developed for dealing with a corpus of repetitions of sequences /aba/, /ada/ and /aga/ uttered by a male French speaker. A predictive coding model was developed to provide guesses about the closure point of the acoustic trajectory /aC/ (with C one of the plosives /b, d, g/) from a given point of the trajectory. We implemented such a model within a Bayesian probabilistic framework, comparing predictions provided by audio-alone inputs with predictions provided by audiovisual inputs.

Importantly, audiovisual inputs were shown to produce better predictions, providing values closer to the actual endpoint than with audio-only inputs. This shows that the visual component provides information able to improve predictions. This toy model is of course highly oversimplified in respect to what should be a reliable system dealing with the whole complexity of speech. However it presents the interest to show that the visual input may strongly improve predictions, in spite of the close synchrony of basic temporal events in the auditory and visual streams, according to the data presented in the Results section. In a word, there is no theoretical requirement for visual lead to argue that visual predictive coding could be at work in the sensory processing of speech in the human brain.

### Concluding remarks

The impressive advances of neurosciences on the processing of speech in the human brain, sometimes simplify the *complexity of speech*, and miss or forget a number of evidence and facts known from long by phoneticians – on the structure of phonetic information, on the auditory and visual cues, on some major principles of speech perception and production. In consequence, there is a serious risk that these advances oversimplify “much of the known complexity of speech as [it] is spoken and of speakers as they speak” [56].

This paper attempts to make clear that the view that vision leads audition is globally oversimplified and often wrong. It should be replaced by the acknowledgement that the temporal relationship between auditory and visual cues is complex, including a range of configurations more or less reflected by the temporal integration window from 30 to 50 ms auditory lead to 170 to 200 ms visual lead.

It is important to recall that fortunately, this caveat does not put in question the experimental studies that capitalized on the presumed “150-ms video lead” to assess audiovisual interactions in EEG or MEG data. Indeed, all these studies (e.g. [4], [5], [7]) used isolated plosive-vowel syllables for which the preparatory visual movement is actually realized without any audio counterpart, hence producing a clear visual anticipation (see Figure 5).

But the pervasive message linking visual lead and visual prediction within a predictive coding stance needs some refinement. Actually, as shown in the last part of this paper, audiovisual predictability does not require audiovisual asynchrony. The development of realistic computational proposals for assessing auditory and audiovisual prediction coding models in speech perception is a challenge for future work in cognitive neuroscience. For this perspective, precise knowledge of the natural statistics of audiovisual speech is a pre-requisite. A number of useful and important data and principles were provided in [21], though the last of its four conclusions needed some refinement. The present paper hopefully contributed to enhance the available knowledge about the complexity of human speech.

## Supporting Information

Figure S1 Trajectories of /ab/, /ad/, /ag/ in the F2–F3 plane.
(TIF)

Figure S2 Variations of lip aperture for /ab/, /ad/, /ag/.
(TIF)

Figure S3 Variations of C_efficiency_ for the 4 prediction models. Mean values in solid lines, maximum and minimum values in dotted lines, for each prediction model (see text).
(TIF)

Text S1 AV predictability without AV asynchrony: a toy model for audio and audiovisual predictive coding of /aCa/ trajectories.
(DOCX)
