# Supplementary figures and images for: No, There Is No 150 ms Lead of Visual Speech on Auditory Speech, but a Range of Audiovisual Asynchronies Varying from Small Audio Lead to Large Audio Lag
Source: PLoS Comput Biol. 2014 Jul 31;10(7):e1003743. doi: 10.1371/journal.pcbi.1003743 (PMC4117430; doi:10.1371/journal.pcbi.1003743)

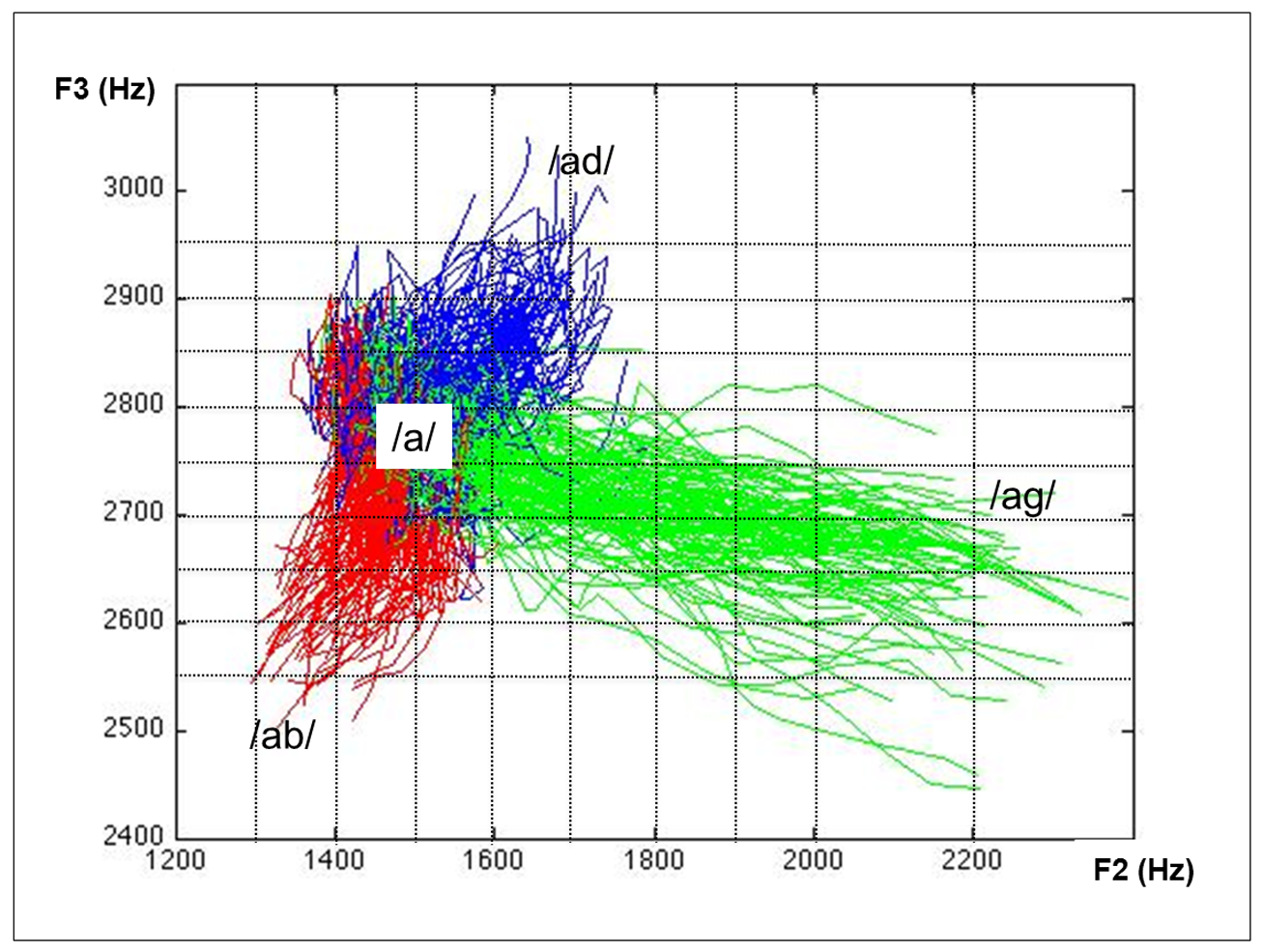

Supplement: Figure S1 — Trajectories of /ab/, /ad/, /ag/ in the F2–F3 plane. (TIF) [file pcbi.1003743.s001.tif]

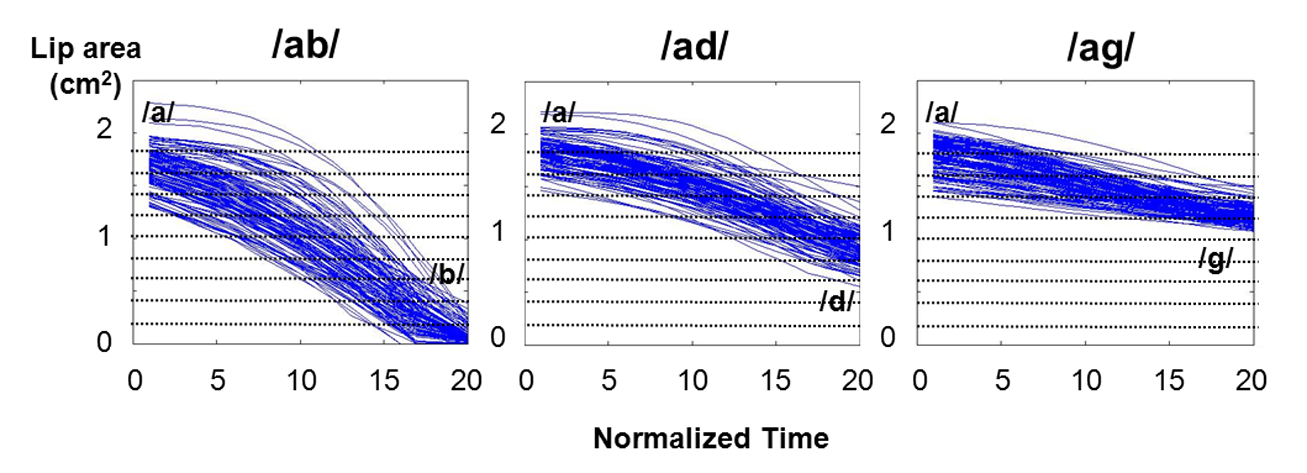

Supplement: Figure S2 — Variations of lip aperture for /ab/, /ad/, /ag/. (TIF) [file pcbi.1003743.s002.tif]

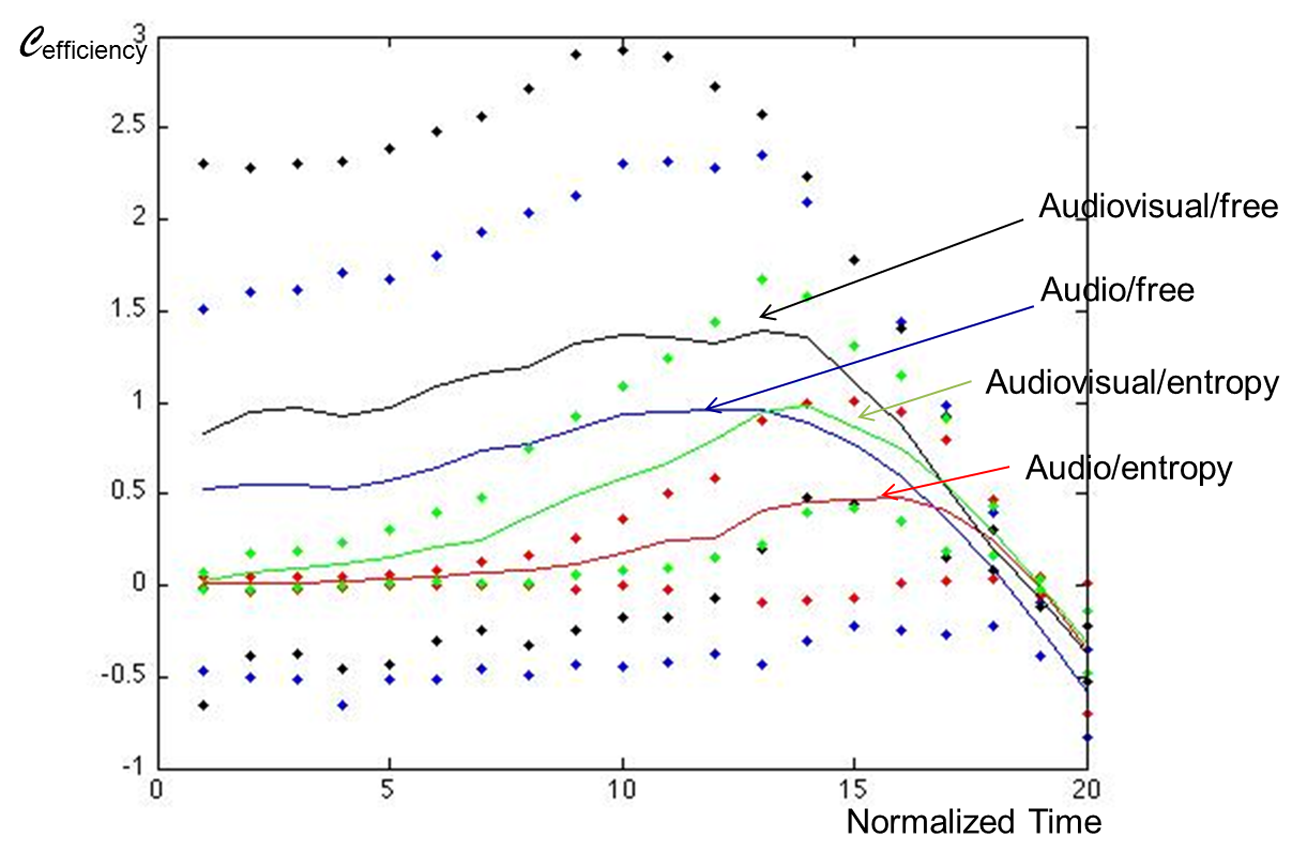

Supplement: Figure S3 — Variations of Cefficiency for the 4 prediction models. Mean values in solid lines, maximum and minimum values in dotted lines, for each prediction model (see text). (TIF) [file pcbi.1003743.s003.tif]
