# Supplementary material for: No, There Is No 150 ms Lead of Visual Speech on Auditory Speech, but a Range of Audiovisual Asynchronies Varying from Small Audio Lead to Large Audio Lag
Source: PLoS Comput Biol. 2014 Jul 31;10(7):e1003743. doi: 10.1371/journal.pcbi.1003743 (PMC4117430; doi:10.1371/journal.pcbi.1003743)
Supplement: Text S1 — AV predictability without AV asynchrony: a toy model for audio and audiovisual predictive coding of /aCa/ trajectories. (DOCX) [file pcbi.1003743.s004.docx]

**Supplementary Text 1**

AV predictability without AV asynchrony: a toy model for audio and audiovisual predictive coding of /aCa/ trajectories

The model was developed for dealing with a corpus of 100 repetitions of sequences /aba/, /ada/ and /aga/ uttered by a male French speaker. The recording and analysis setup was the same as the one described in the Methods section. For each /aC/ item (C within /b d g/), formants F2 and F3 were extracted and rescaled in time so that each /aC/ trajectory was described by a temporal sequence with 20 points, that is F2(1:20) and F3(1:20) (Figure S1). Similarly, the values of lip aperture L were automatically extracted, the same temporal extraction and rescaling process was applied, and the lip trajectory for each /aC/ utterance was described by a temporal sequence with 20 points, that is L(1:20) (Figure S2).

From these trajectories, a predictive coding model was developed to provide guesses about the final point of the acoustic trajectory from a given point of the trajectory. We implemented such a model within a Bayesian probabilistic framework. For this aim, we first discretized the (F2, F3) space into 100 values with 10 values for F2 and 10 for F3 sampling the acoustic space in Hz (see Figure S1). We also discretized L with 10 values regularly sampling the lip opening space in cm (see Figure S2). Then, from the 20 points in time of the 100 repetitions of the 3 types of stimuli (6000 points altogether) a joint audio probability function p_A_(C, C_final_) was learnt. This is the probability to be in position C (from 1 to 100) at a given time and then in position C_final_ at the end of the trajectory. An audiovisual probability function p_AV_(C, L, C_final_) was also learnt, that is the probability to be in position (C, L) at a given time and then in position C_final_ at the end of the trajectory.

From these probability functions two audio prediction models were elaborated:

- Model A_free_, for which the prediction about final position C_pred_ when the system is in C is provided by:

C_pred/Afree_ = E(C_final_ / C) α Σ_Cfinal_ C_final_ p_A_(C, C_final_)

This model just computes the mean possible final position knowing the set of probability values p_A_(C, C_final_).

- Model A_entropy_, for which the previous prediction is used under the constraint that entropy of the distribution of possible C_final_ positions stays at a low value (with a criterion using the sum of variances of possible F2 and F3 values of C_final_ positions). This means that if in a given position the possible C_final_ positions are too dispersed (as it is the case for example at the beginning of a trajectory around /a/) the C_pred/Afree_ is not used and the true prediction stays at the position of the actual position C.

C_pred/Aentropy_ = w C_pred/Afree_ + (1-w) C with w=f(entropy)

In this model, if entropy is low, C_pred/Aentropy_ is close to C_pred/Afree_, and if it is high, C_pred/Aentropy_ is close to C.

Audiovisual prediction models are introduced in the same way:

- Model AV_free_, for which the prediction about final position when the system is in C is provided by:

C_pred/AVfree_ = E(C_final_ / C, L) α Σ_Cfinal_ C_final_ p_AV_(C, L, C_final_)

- Model AV_entropy_, with:

C_pred/AVentropy_ = w C_pred/AVfree_ + (1-w) C with w=f(entropy)

To evaluate these prediction models we estimated the “efficiency” of their prediction. Efficiency was provided by computing the difference between the distance between actual position and true final position, and the distance between predicted position and true final position:

d(C, C_final_) - d(C_pred_, C_final_)

A positive difference expresses the fact that prediction is closer to final position than is actual position: the prediction is “efficient” in this case, and not if the difference is negative. C_efficiency_(t) is computed for each normalized time between 1 and 20 as the mean of this difference for all trajectories.

On Figure S3 we display C_efficiency_ for the 4 prediction models with mean values (in solid lines) and minimum and maximum values (in dotted lines). It appears that “free” predictions produce larger mean values than “entropy” predictions but with a number of negative values, which show that predictions can be wrong, particularly at the beginning of the trajectory. On the contrary, “entropy” models almost never produce wrong predictions: most values are above zero.

Importantly, audiovisual predictions produce larger values than auditory prediction, particularly for “entropy” models. This shows that the visual component improves predictions. This toy model is of course highly oversimplified in respect to what should be a reliable system dealing with the whole complexity of speech. However it presents the interest to show (1) how predictions can be made, (2) how their efficiency can be controlled thanks to an entropy-based criterion, (3) how they evolve in time in these configurations and (4) most importantly, that the visual input may strongly improve predictions, in spite of the close synchrony of basic temporal events in the auditory and visual streams, according to the data presented in the Results section. The predictions summarized by the display in Figure S3 concern chained sequences similar to those analyzed in this paper – for which there is almost no visual anticipation on the auditory stream according to Figure 7. Of course, for isolated syllables the situation would be quite different: the closing labial gesture would provide a prediction while the lack of sound would lead to no audio prediction at all, hence the gain provided by the visual input would be trivial.

The prediction models presented in this paper are obviously preliminary and simplistic, and assessed on very stereotyped speech sequences. More powerful techniques will have to be explored to attempt to rescale this study towards more realistic corpora. The use of entropic predictions, as introduced in this paper, will be an important challenge for future studies in this framework.
